# Comment on ‘Hand pattern indicates risk of prostate cancer’

**DOI:** 10.1038/bjc.2011.232

**Published:** 2011-07-26

**Authors:** M Cheema, A Sharif

**Affiliations:** 1 Department of plastic surgery, Norfolk and Norwich University Hospital NHS Trust, UK; 2 GPVTS, Black Country rotation, West Midlands, UK

**Sir,**

We read with much interest the online article by Rahman *et al* (2011) and congratulate the authors on completing this large case–control study. This study concludes that self-reported ratio of second and fourth digit (2D : 4D ratio) negatively correlates with risk of prostate cancer. We have some reservations about the study.

- The investigators agree that self-reported digit lengths show poor agreement with those measured by researchers (Manning *et al*, 2005; Caswell and Manning, 2009). Their rationale for using this method is that a larger study population may provide that correlation. It may have been better to achieve that correlation first, rather than carry out a study based on an unverified outcome.
- Digits continue to grow as the skeleton matures. In the absence of evidence that 2D : 4D ratio is preserved from neonates to adults, it is not possible to conclude that adult proportions reflect prenatal hormone levels. Even with that evidence, the difference in digit length in adults may well reflect environmental factors or even difference in dexterity or pattern of hand usage amongst the individuals.
